# Estimating Animal Abundance in Ground Beef Batches Assayed with Molecular Markers

**DOI:** 10.1371/journal.pone.0034191

**Published:** 2012-03-30

**Authors:** Xin-Sheng Hu, Janika Simila, Sindey Schueler Platz, Stephen S. Moore, Graham Plastow, Ciaran N. Meghen

**Affiliations:** 1 Department of Agricultural, Food and Nutritional Science, University of Alberta, Edmonton, Canada; 2 IdentiGEN North America, Lawrence, Kansas, United States of America; 3 Centre for Animal Science, Queensland Alliance for Agriculture and Food Innovation, The University of Queensland, St Lucia, Australia; Pennsylvania State University, United States of America

## Abstract

Estimating animal abundance in industrial scale batches of ground meat is important for mapping meat products through the manufacturing process and for effectively tracing the finished product during a food safety recall. The processing of ground beef involves a potentially large number of animals from diverse sources in a single product batch, which produces a high heterogeneity in capture probability. In order to estimate animal abundance through DNA profiling of ground beef constituents, two parameter-based statistical models were developed for incidence data. Simulations were applied to evaluate the maximum likelihood estimate (MLE) of a joint likelihood function from multiple surveys, showing superiority in the presence of high capture heterogeneity with small sample sizes, or comparable estimation in the presence of low capture heterogeneity with a large sample size when compared to other existing models. Our model employs the full information on the pattern of the capture-recapture frequencies from multiple samples. We applied the proposed models to estimate animal abundance in six manufacturing beef batches, genotyped using 30 single nucleotide polymorphism (SNP) markers, from a large scale beef grinding facility. Results show that between 411∼1367 animals were present in six manufacturing beef batches. These estimates are informative as a reference for improving recall processes and tracing finished meat products back to source.

## Introduction

Estimating animal abundance in manufactured batches of fresh ground meat is an important phase in traceability and certification in meat supply chains [1].This is of a particular value in the event of a microbial contamination incident given that fresh ground beef accounts for more than 40% of all beef consumed in Canada [2] and 42% in the United States [3]. To identify whole muscle meat products, such as steaks and joints, throughout the supply chain, DNA profiling is currently applied through the use of reference animal or carcass databases, analogous to the DNA databases widely used in human forensics. In a large scale industrial manufacturer, a single ground beef batch may consist of many hundreds of animals from diverse sources, which may include more than one country of origin. Characterizing the distribution of these individuals in large grind batches informs the possibility of developing a recall management tool based on DNA profiling. Estimating animal abundance has been widely applied in ecology and wild life conservation [4], [5], [6], [7]. However, the mixture in ground beef batches complicates the application of this technique, including isolation of individual DNA profiles and the selection of an appropriate statistical model. The objective of this study is to focus on the statistical model for a preliminary estimate of animal abundances in grind meat batches, given the heterogeneity arising from different manufacturing systems and the absence of a reference DNA profile database.

We employ the conventional mark-recapture methodology to estimate animal abundance, with multiple surveys in individual manufacturing batches for estimating capture and recapture frequencies. Samples are taken from the finished ground beef batch and individual animal contributors identified by subdividing the sample into constituent discrete muscle fibres for DNA extraction and single nucleotide polymorphisms (SNP) genotyping [1]. Matching DNA profiles among samples, analogous to the case of sampling with replacement, are used to estimate recapture frequency. Two specific features are crucial for statistical modelling in ground meat batches. One is the presence of a highly heterogeneous capture probability among individuals in a single batch. This can arise where an unequal amount of useable carcass from distinct animals is blended into individual batches for ground beef. This forms the biological basis for generating unequal capture probability among distinct animals. The other is that the number of animals in a single beef batch could be very large in industrial scale manufacturing. This can result in a large number of animals not being captured or captured at a low frequency, in addition only a few animals may be captured at a relatively high frequency. These two features limit the suitability of most existing models for estimating population size in ground meat batches.

Methodologically, many statistical models have been developed using the mark-recapture framework for population size estimation, including the non-parameter and parameter estimators, the models for equal and unequal capture probability, and the models for discrete- and continuous-time surveys (for comprehensive reviews, see [5], [6], [7]). The well-known non-parameter estimators include Lincoln-Petersen's estimator, the jackknife estimator [8], [9], the bootstrap estimator [9], the moment estimator [10], [11], and the sample-coverage (SC) estimator [12]. Most non-parameter estimators underestimate population size when a small proportion of animals are captured. The jackknife estimator can produce appropriate estimates when many individuals are captured multiple times [13]. Chao's estimator (Chao-1) performs well for a lower level of heterogeneity in capture probability or when a majority of individuals are captured [10]. Xu et al. [11] recently proposed an alternative non-parameter estimator that slightly modifies Chao-1 estimator using a different moment approach. The commonality is that these estimators (except the high-order jackknife and SC estimator) mainly employ partial information on the observed capture and recapture frequencies in multiple surveys.

With a reference to the parameter-based estimators, a few methods have been developed to derive maximum likelihood estimate (MLE) of population size since Fisher's logarithm series model [7], [14], [15], [16]. These methods are mainly based on the abundance data (frequency count) although connections are available for a few abundance and incidence models [17]. Crucial to the parameter-based methodology is to select an appropriate function to describe the pattern of capture-recapture frequencies. Chao and Bunge [18] used a Gamma-mixed Poisson or negative binomial distribution to derive MLE. Shen and He [19] used a modified beta function to derive MLE for species richness. The commonality is that these methods employ the full information on the pattern of capture-recapture frequencies. These methods have limited performance when the heterogeneity in capture probability is large or when most individuals are not captured in multiple surveys. This motivated us to develop alternative estimators that are suitable for the population with a high heterogeneity in capture probability.

We developed two parametric models for incidence data to estimate population size: Model I is based on a function similar to a modified continuous version of Fisher's logarithm series model, which can deal with the population with a high heterogeneity in capture probability; Model II is a modified beta function, with an alternative zero-truncated function to the modified function of Shen and He [19]. Model II can deal with the population with a relatively low heterogeneity in capture probability. In the following sections, the proposed models are described, including the detailed procedure of deriving MLE. The proposed estimators are then compared with other existing non-parameter estimators through simulations with different survey schemes and the use of previously published empirical datasets. Finally, we apply the proposed models to estimating the number of animals in six manufacturing beef batches, each of approximately 1 metric tonne in weight, genotyped with 30 SNP markers, selected for identification [20]. Inferences on population sizes in each batch of fresh ground beef are drawn from comprehensive analyses with multiple estimators.

## Methods

### The Model and Estimator

We begin by briefly summarizing Burnham and Overton's model and then proceed to propose an alternative method to estimate population size. Consider a closed population with constant *N* unique individuals that are indexed by 1, …, and *N*. There are *t* surveys through non-invasive genetic samples (analogous to the sampling with replacement). Let 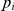 (*i* = 1, 2, …, *N*) be the capture probability of the *i*th individual at each survey (constant capture probability assumption). Here, we assume that the capture probability for each individual is nonzero at each survey (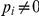) and that unequal capture probabilities exist among different individuals, i.e. 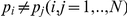. The capture probabilities, 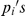, are a random sample from a probability density distribution 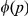. Note that 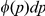 is equivalent to the notation 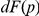 of Burnham and Overton [8]. Like previous studies [10], the multiple samples can be arranged in a 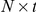 matrix 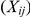 (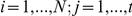) where 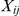 is the observed frequency of the *i*th individual in the *j*th survey. Let *n* be the total number of observed distinct individuals caught in the *t* samples, which can be expressed as 
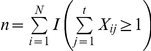
 where *I*(*A*), the indicator function, is equal to 1 when event *A* occurs and 0 otherwise. Let 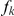 be the number of individuals captured exactly *k* times (*k* = 0, 1,…, *t*) in the *t* samples, which can be expressed as 
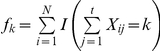
 ([10], p 784). Thus, we get the captured individuals in total, 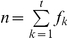, and the population size *N* is 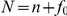 where *f*_0_ is the number of individuals that are not captured in the *t* samples.

According to Burnham and Overton [8], the joint likelihood function for the whole *t* samples can be expressed as
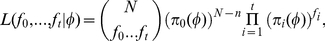
(1)where 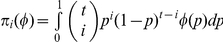. 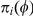 is the probability for the *t* samples with *i* unique individuals in the multinomial distribution. The integration in 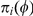 removes the impacts of a random sample of 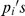. Based on the above general framework, Burnham and Overton [8] developed a *k*th-order jackknife estimator for population size *N*. Using the same framework, Chao [10] developed an alternative non-parameter estimator (moment estimator) of *N*. Here, we proceed with the same framework to develop an unconditional MLE of *N* by hypothesizing two different types of capture probability density distributions 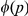.

Since a non-zero capture probability for each individual (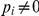, *i* = 1,…, *N*) is assumed at each survey, the zero point as the lower bound must be eliminated in calculating probability 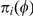. In the absence of prior information about individual capture probabilities, it is difficult to determine the exact capture probability 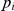 and probability density function 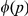 [21]. Biologically, different sources of uncontrollable and unobservable variations can generate heterogeneity in capture probability among individuals or the relative occurrences of different individuals at each survey. This variation may arise from behavioural difference among individuals or different foraging areas or different exposures to traps [21]. How to determine such impacts on the capture probability density distributions 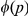 remains to be explored. In this study, we consider two capture probability density distributions that are suitable for a large population.

**Figure 1:**
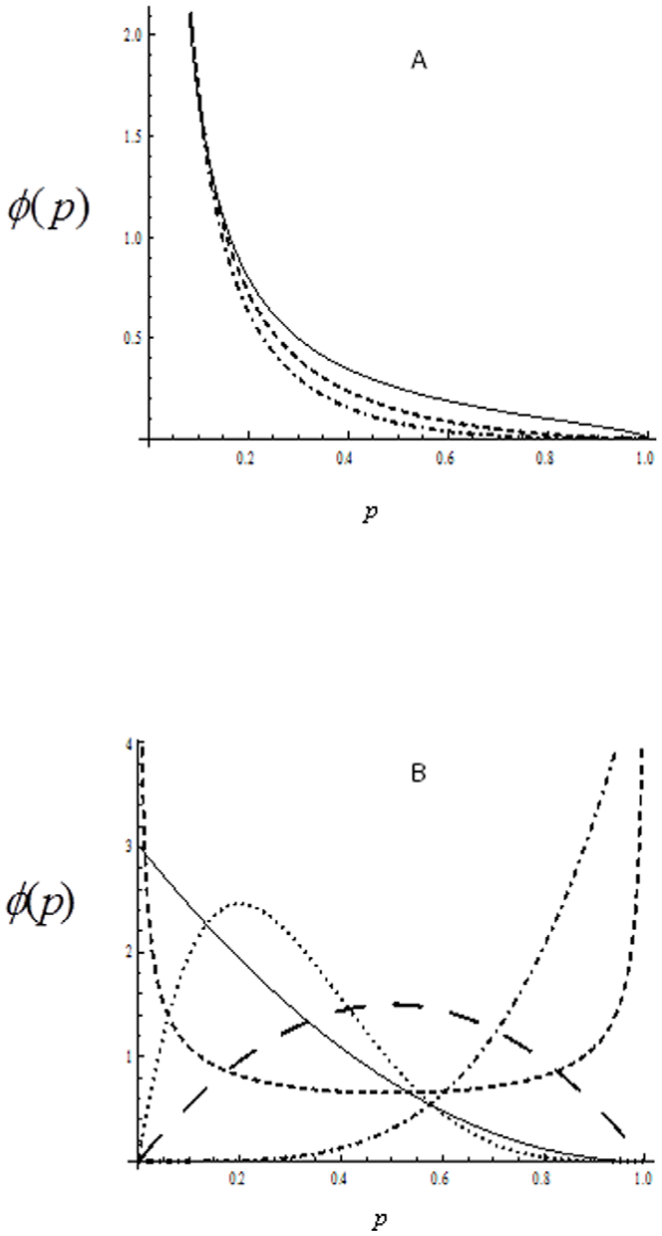
The distribution of capture probability density. A: Capture probability density function (pdf) for Model I, 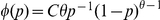, given a population size *N* = 500: line for *θ* = 1.5, dashed line for *θ* = 2.5, and dot dashed line for *θ* = 3.5. The skew of the capture probability distribution increases as the parameter *θ* increases. B: Capture probability density function (pdf) for Model II, 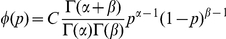, given a population *N* = 500: line for *α* = 1, *β* = 3.0; dotted line for *α* = 2, *β* = 5 (skewed bell-shape); thick dashed line (bell-shape) for *α* = 2, *β* = 2; dashed line (U-shape) for *α* = 0.5, *β* = 0.5; and dot dashed line for *α* = 5, *β* = 1.0. An array of capture probability distributions can be generated by changing parameters *α* and *β*.

In Model I, we assume that the probability density function (pdf), 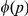 for a capture probability *p* has the following expression:
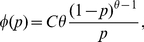
(2)where 
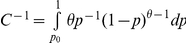
, the total number of captures given the minimum non-zero capture probability 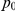, and 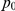 is the lower bound of capture probability. The biological meaning of parameter *θ* (>1) is termed as the average capture change per individual per unit time. This setting is based on the biological phenomenon that the observed abundance distribution, 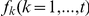, frequently exhibits an “opposite J-shape” pattern (Figure 1A). Many individuals are captured once and a few individuals are captured more than once. 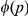 can be used to describe the phenomena in mark-recapture experiments where the number of captured individuals decreases with the capture probability *p* (without a long tail of frequency distribution).

Several considerations are needed in setting 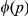 in Eq. (2). First, the proper pdf 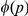 is derived by normalizing the function 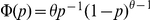 by considering *p* as the capture probability for incidence data rather than the relative frequency for abundance data (e.g., allele frequency in a population or the relative species abundance in a community; [22]). Here, we borrow the function 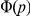 from the neutral theory (the infinite number of allele model) in molecular population genetics [23], [24]. 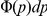 is the expected number of unique individuals whose capture probabilities (*p*) fall within the range (*p*, *p*+*dp*) and 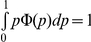. In population genetics, the function 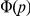 is the well known function for describing the abundance distribution of neutral alleles in a closed population, where *θ* is the average number of alleles generated by mutation per generation. Again, the conceptual difference is that *p* is not the gene frequency (abundance data) but the capture probability (incidence data) in this study. The capture distribution for an array of capture probabilities is analogous in distribution pattern to but different in biological meaning from the abundance distribution of an array of gene frequencies [24], [25] (pp. 205–206). Second, for the abundance model, 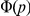 (not pdf) is the same as the well-known Fisher's logarithmic series (discrete) distribution [14] except that 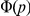 is the version for a continuous distribution ([26], p 250). Leigh ([27], Appendix 8.2) transformed Fisher's logarithm series into 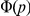. Fisher's *α* parameter in the logarithm series function is analogous to *θ* here, which is also analogous to Hubbell's *θ* in describing the pattern of species richness and relative abundances in a neutral metacommunity (the fundamental biodiversity parameter; [22], [28]). Chao and Bunge [18] also employed this kind of function (gamma-mixed Poisson) to derive the probability for the *t* samples with *i* unique individuals for the abundance data, analogous in concept to but different in expression to 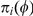 here. In this study, 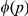 in Eq. (2) for the incidence data can be seen as the model similar to the zero-truncated continuous version of Fisher's logarithmic series model. Third, the lower bound 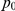 for an individual capture probability must be nonzero in biology except for the case of extinction, although a zero bound is allowed from the statistics point of view. One feature of the function 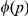 is that its integration value becomes substantially large as 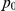 becomes smaller, given a constant population size ([25], p 210). How to determine the lower bound remains to be explored in biology. In practice, it is difficult to even catch the individual with the capture probability of 1%. One way is to directly estimate 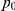 by considering 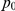 as one additional parameter. However, extensive simulations indicate that this consideration leads to the difficulty of obtaining convergent estimates (results not shown here). In the following parts, we set 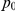 = 1/*N*, and this lower bound becomes sufficiently small (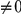) as the population size increases. Thus, Model I with 1/*N* as the lower bound is suitable for a large population. It is noteworthy that, for the abundance data, a setting similar to the above but with different biological meanings exists in population genetics ([25], p 210; [29], p 398) or in community ecology [22], [30], where 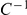 represents the total number of existent alleles in a population or existent individuals in a metacommunity, respectively.

Since a non-zero capture probability is considered for each of *N* individuals in the population, the lower bound in 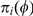 is correspondingly changed, i.e.
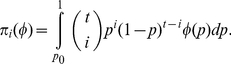
(3)Here, 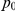 in 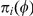 is set as 1/*N*, and the model is suitable for a large population. The sum of 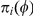 remains 1, i.e. 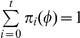.

The general likelihood function can be decomposed into two sub-likelihood functions [15], [16], [18], [19], i.e. 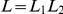 where 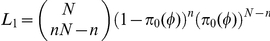 and 
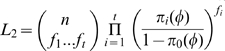
. The difference from previous models is that each sub-likelihood function (*L_1_* or *L_2_*) is the function of two parameters (*N* and *θ*). Calculation of conditional MLE remains to be explored. To derive the MLE of *N* and 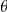, we simply use the global likelihood function instead of decomposing it into two different components. Like Stollenwerk and Jansen ([31], pp 185–191), we approximate the population size *N* as a continuous variable in derivation.

Let 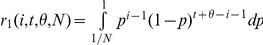, 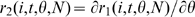, and 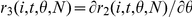. 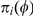 can be expressed as 
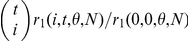
. Let 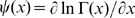, the digamma function 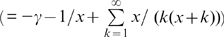, and 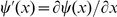, the trigamma function (
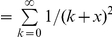
)) [32]. Note that the first term in 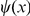 is Euler's constant *γ* = 0.5772156649. The first- and second-order partial differentials of the log likelihood function 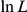 with respect to *N* and 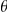 are derived in Appendix S1. Population size *N* and the parameter 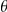 can be solved using Newton and Raphson's iterative method (with a fast convergent speed):
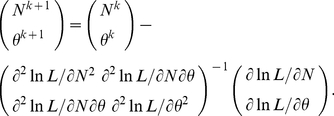
(4)


The initial values for *N* and 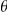 in iteration can be set as *n* and 0, respectively. Iterative calculations are continued till convergence for each estimate is achieved. Note that no failure convergence existed in all simulations described in the next section. The variances for estimates *N* and 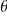 can be calculated from the diagonal elements of the inverse variance-covariance matrix (inverse of Fisher's information matrix) at convergence: 
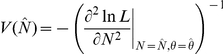
 and 
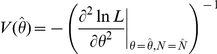
.

In Model II, 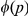, is set as a zero-truncated beta distribution function:
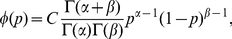
(5)where 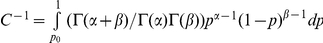 (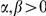). The biological meanings of parameters *α* and *β* are termed as the average capture changes per individual per unit time for individuals with capture probabilities *p* and 1−*p*, respectively. This type of capture probability density function, similar to Pearson's Type I model ([26], p 248), can be used to represent a variety of patterns of 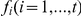 distributions under different parameter settings, including the opposite J-shape pattern (Figure 1B). The difference from Model I is that the pattern for the capture-recapture frequencies generated by Model II is not as highly skewed as that generated by Model I, i.e. a relative lower heterogeneity in capture probability. When 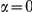, Model II reduces to Model I. When 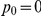, Model II reduces to the model of a beta-binomial distribution mixture [21]. Shen and He [19] recently also employed the beta function to describe species richness distribution, but used a different zero- truncated transformation by changing 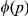. One constraint in Shen and He's model is that the setting of 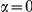 can lead their constant 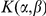 to an infinite value, violating the condition in setting their 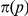 (equivalent to 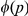 here). Again, 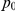 in 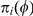 and 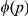 is set as 1/*N*. Thus, Model II is suitable for a large population.

Like in Model I, Eq. (3) remains unaltered after changing the lower bound in 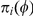 by 1/*N*. To derive MLE, let 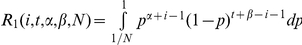, 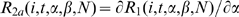, 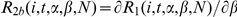, 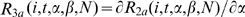, 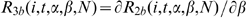, and 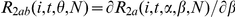. 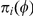 can be expressed as 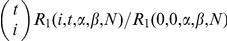. The first- and second-order partial differentials of the log likelihood function 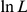 with respect to *N*, 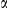, and 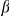 are derived in Appendix S2. Similarly, these three parameters can be estimated using Newton and Raphson's iterative method:
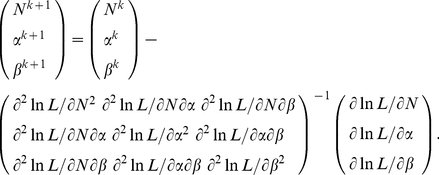
(6)


The initial values during the iterative calculation can be set as *n*, 0, and 0 for *N*, 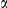, and 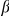, respectively. Iterative calculations are continued till convergence for each parameter. Note that non convergence can occur under some parameter settings, such as the case of *α* = 1 and *β* = 3.0 in simulations described in the next section. The variances for estimates *N*, 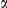, and 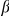 can be calculated from the diagonal elements of the inverse variance-covariance matrix at convergence.

### Monte Carlo Simulations and Comparisons

#### Simulation Data Generation

To examine the properties of the proposed models, we analyzed several sampling schemes based on the distribution pattern of 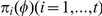, generated by different parameter settings in capture probability density function 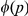. The aims are (i) to look at the impacts of different sampling schemes (the number of surveys) under a known population size *N* and parameters, and (ii) to look if some non-parameter estimators perform well with the capture probability distribution assumed in Models I and II since estimates of population size are sensitive to the assumption of 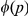 [21]. Similar to Shen and He [19], three non-parameter estimators were selected: the first-order jackknife estimator [33], 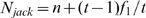, the bootstrap estimator [9], 
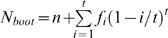
, and Chao-1 estimator [10], 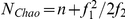. The jackknife and Chao-1 estimators only employ partial information of capture-recapture frequencies; while the bootstrap estimator employs the full capture-recapture frequencies in the *t* surveys in a way different from the proposed models. These three non-parameter estimators have been extensively assessed in previous studies from the literature.

Given the population size *N*, the setting for a sample size is constrained by the fixed sum ( = *N*) of the observed unique individuals in total and the unobserved individuals. An arbitrary setting of sample size *n* could result in the total population size exceeding *N* according to the distribution 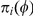. Thus, the simulated samples for the proposed two- and three-parameter models are generated in the following steps. Given a population size *N*, *t* surveys, and parameter 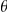 for Model I, or parameters *α* and *β* for Model II, calculate each probability 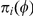 (*i* = 0,1,…, *t*). Then, use these probabilities (multinomial distribution) to generate the numbers of individuals with different capture-recapture frequencies 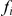
's (*i* = 0, 1, …, *t*; 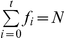 ). Note that the samples of capture-recapture frequencies, generated by this way are equivalent to those generated by Otis et al.'s [13] method that is based on assigning each individual a certain capture probability based on 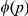. The routine of Press et al. ([34], pp 210–211) was used to generate random numbers with uniform distribution within (0, 1) for sampling purpose. The observed frequencies, 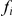
's (*i* = 1,…,*t*), were then used to estimate parameters according to Eq.(4) for Model I and Eq. (6) for Model II. We consider that the convergence is reached when the absolute difference between two consecutive iterative values is less than 10^−5^ for each parameter although an even smaller number can be set at the expense of long-time iterations. Three non-parameter estimators were also calculated from the observed 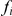
's (*i* = 1,…, *t*). One hundred independent data sets were created, and each was used to estimate all parameters. Means and standard deviations (*S_d_*) of estimated parameters were calculated from these replicated datasets. The standard deviations for *N*, *θ*, *α*, and *β* were also calculated from averaged Fisher information index, in addition to empirical standard deviations.

Several sampling schemes were simulated in Model I, with the number of surveys increasing from 2 to 10 under three different patterns of capture probability distributions (*θ* = 1.5, 2.5, and 3.5; Figure 1A). The distribution becomes more skewed as parameter *θ* increases from 1.5 to 3.5. In Model II, five different patterns of capture probability distributions were simulated (Figure 1B): *α* = 1, *β* = 3 (opposite J-shape); *α* = 2, *β* = 5 (skewed bell-shape); *α* = 2, *β* = 2 (bell-shape); *α* = 0.5, *β* = 0.5 (U-shape); and *α* = 5, *β* = 1 (J-shape) for the known parameter settings. These distribution patterns may occur for the capture-recapture frequencies in different animal species in trapping experiments or for plant species in spatiotemporal quadrat surveys in ecology. Four sampling schemes were simulated in each of the five patterns, with the number of surveys increasing from 4 to 10. Programs in *C* are available upon request from Hu.

### Simulation Comparisons

In Model I, the average estimates of population size 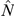 and parameter 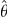 in each of the three capture frequency distributions are generally in good agreement with their actual values (Table 1). The actual population size *N* and parameter *θ* are within the ranges of one standard deviation of estimates in each case. The standard deviations for 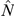 and 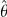 calculated from the inverse of the Fisher information matrix (not shown in Table 1) are consistent with the empirical values for a large sample size (*n*). Generally, the standard deviations for each parameter estimate decrease as the number of surveys increases. Based on the distribution of probability 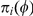, the average number of sample size per survey (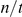) decreases as the number of surveys increases from *t* = 2 to 10. The observable sample size in total (*n*) generated from the probability distribution (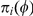, *i* = 1,…, *t*) decreases as the capture probability distribution 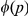 becomes more skewed (*θ* changing from 1.5 to 3.5; Figure 1A). The results indicate that the combination of more surveys with a small sample size per survey can produce better estimates than the combination of a small number of surveys with a large sample size per survey (Table 1). The three non-parameter estimators substantially underestimate population size *N* although the average estimates of population size increase with an increased number of surveys (detailed data not shown here). When the capture probability distribution 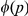 becomes more skewed, the non-parameter estimators produce severe underestimates of *N*. Standard deviations exhibit different patterns for different non-parameter estimators, but each is related to the extent of skewness of the capture probability distribution. Thus, these non-parameter estimators are not suitable for the population with the capture probability distribution 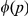 assumed in Model I where a high heterogeneity of capture probability exists [35].

**Table 1:** Mean estimates and their standard deviations of Model I under different parameter settings.

Cases	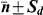	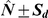	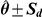
*θ* = 1.5, *N* = 500			
*t* = 2	93.74±7.68	510.71±121.31	1.58±0.46
4	140.24±10.61	512.49±69.63	1.56±0.25
6	169.72±10.45	498.98±45.39	1.49±0.20
8	191.70±10.76	504.93±44.81	1.54±0.18
10	209.08±11.58	502.61±39.52	1.53±0.17
*θ* = 2.5, *N* = 500			
*t* = 2	69.18±7.37	572.45±218.27	2.95±1.28
4	107.59±7.76	504.87±77.30	2.64±0.51
6	138.91±10.16	511.75±67.41	2.59±0.42
8	160.26±10.72	504.08±54.24	2.56±0.38
10	177.88±10.52	504.80±44.97	2.60±0.35
*θ* = 3.5, *N* = 500			
*t* = 2	54.52±6.79	584.84±352.66	4.37±3.28
4	90.96±8.43	520.36±98.50	3.80±0.85
6	117.62±9.28	496.36±77.58	3.57±0.68
8	139.48±10.16	502.29±57.80	3.64±0.57
10	160.15±10.20	510.78±53.21	3.60±0.50
*θ* = 1.5, *N* = 1000			
*t* = 2	168.82±11.65	1027.95±182.73	1.56±0.31
4	245.90±11.89	993.45±91.11	1.53±0.18
6	301.76±15.12	1017.68±78.31	1.56±0.15
8	341.80±13.74	1009.77±68.05	1.53±0.13
10	373.13±16.84	1003.89±63.04	1.52±0.14
*θ* = 2.5, *N* = 1000			
*t* = 2	119.85±9.95	1047.93±270.27	2.69±0.74
4	193.62±12.32	1033.99±133.52	2.61±0.43
6	240.66±12.99	995.56±106.77	2.54±0.34
8	280.63±16.08	1004.20±84.92	2.57±0.29
10	314.34±14.89	1012.70±74.53	2.58±0.23
*θ* = 3.5, *N* = 1000			
*t* = 2	95.58±10.03	1039.75±336.73	3.72±1.29
4	162.24±11.58	1057.76±176.93	3.71±0.69
6	207.03±12.98	1006.41±105.71	3.58±0.49
8	247.82±13.41	1022.14±91.10	3.59±0.41
10	277.06±14.47	1007.08±88.68	3.56±0.39

Simulation results were obtained from 100 independent runs*.

* : 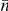: the average sample size for the *t* surveys; 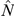: the average estimate of population size; 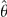: the average estimate of parameter θ; 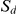: the standard deviation.

With Model II, the average estimates of *N*, *α*, and *β* become closer to the actual values as the sampling scheme changes from *t* = 4 to 10 in each of the five capture probability distributions (Table 2). The actual population size and parameters (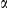, and 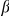) are within the ranges of one standard deviation of estimates in each case. Again, the standard deviations of each parameter (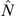, 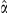, and 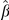) calculated from the inverse of the Fisher information matrix (not shown in Table 2) are very close to the empirical values. The standard deviations for each parameter estimate decrease as the number of surveys increases from *t* = 4 to 10. The average observed sample sizes (*n*) are closely related to the capture probability distribution 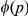 and exhibit considerable variation among the five distributions. A trade-off relationship does not exist between the number of surveys and the average number of individuals captured per survey. In each case, the standard deviations for observed sample sizes decrease as the number of surveys increases from *t* = 4 to 10. Given a sampling scheme, the observable sample size in total (*n*) is the smallest in the case *α* = 1 and *β* = 3, but the largest in the case of *α* = 5 and *β* = 1 (Figure 1B).The observable sample size in total (*n*) reaches the maximum in the case *α* = 5 and *β* = 1 since almost all individuals can be captured in this distribution (Figure 1B).

**Table 2:** Comparison of the proposed three-parameter model with three existing non-parameter estimators (the true population size *N* = 500, and 100 independent simulations).

Cases	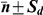	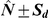	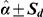	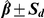	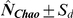	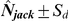	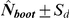
*α* = 1, *β* = 3							
*t* = 4	283.76±9.53	448.21±73.77	1.88±0.96	4.50±1.77	395.72±23.81	388.15±14.60	333.44±11.57
6	336.97±10.09	486.92±48.70	1.22±0.38	3.35±0.72	419.90±20.30	439.56±15.53	387.34±13.85
8	364.45±9.66	503.40±41.32	1.05±0.29	3.16±0.57	437.71±19.41	461.94±14.41	413.01±11.18
10	387.67±10.04	496.06±33.13	1.11±0.26	3.22±0.50	446.74±17.96	474.24±15.38	431.93±10.20
*α* = 2, *β* = 5							
*t* = 4	332.68±11.00	465.43±39.80	4.38±3.93	9.10±7.00	451.53±22.52	453.58±16.18	390.77±13.12
6	387.21±9.54	500.93±39.61	2.37±0.90	5.77±1.75	472.74±23.28	502.04±16.02	444.57±11.76
8	416.98±8.44	496.26±23.42	2.27±0.62	5.61±1.25	475.62±15.92	513.26±13.57	467.52±10.25
10	435.58±7.32	496.74±14.64	2.12±0.41	5.30±0.85	479.67±12.68	516.17±10.99	479.53±8.24
*α* = 0.5, *β* = 0.5							
*t* = 4	371.65±11.02	492.16±45.44	0.59±0.24	0.53±0.10	416.47±16.47	431.40±13.82	401.74±12.03
6	396.87±8.45	492.12±27.83	0.55±0.12	0.52±0.06	436.41±14.42	449.47±11.44	423.45±9.46
8	413.72±7.58	504.01±28.36	0.51±0.13	0.51±0.06	451.14±15.18	462.69±10.92	438.41±8.45
10	422.94±7.04	499.84±19.17	0.51±0.11	0.51±0.05	452.48±12.85	465.81±9.60	445.05±7.66
*α* = 2, *β* = 2							
*t* = 4	427.05±7.69	495.21±22.99	2.30±0.83	2.20±0.63	477.43±14.04	511.45±11.96	471.10±9.05
6	457.85±5.74	496.60±11.54	2.21±0.43	2.17±0.35	486.68±10.57	516.49±9.47	490.87±6.52
8	472.51±4.83	498.89±8.84	2.09±0.33	2.08±0.29	491.89±8.12	515.27±8.21	497.67±5.77
10	480.13±4.51	497.53±5.79	2.13±0.24	2.11±0.23	493.50±6.14	510.97±6.13	499.08±4.73
*α* = 5, *β* = 1							
*t* = 4	496.12±1.95	499.26±2.43	6.01±2.08	1.17±0.38	499.42±2.55	510.32±4.04	506.39±2.37
6	499.07±0.99	499.75±1.20	4.81±0.79	0.97±0.15	500.21±1.34	503.95±1.99	503.29±1.32
8	499.40±0.70	498.33±0.70	3.92±0.46	0.88±0.11	499.82±0.93	500.80±1.39	501.07±0.80
10	499.91±0.29	499.46±0.30	5.21±0.70	1.03±0.13	500.19±0.76	500.54±0.81	500.72±0.46

Unlike the results in Model I, Model II has a comparable performance to the non-parameter estimators in four of the five types of distributions, the exception being *α* = 1 and *β* = 3, where underestimates are obtained (Table 2). The scheme with more surveys can produce better estimates in each case. The results indicate that the three non-parameter estimators generally perform well for the capture probability distribution 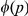 assumed in Model II.

### Comparisons Using Published Empirical Data Sets

Here, we use two published datasets to demonstrate the application of the proposed models. The first example is the well-known Fisher's butterfly data that was collected in Malaya [14]. The paper provided the observed distribution of frequencies of butterflies for species abundance ranging from 1 to 24 ([14], p 43). This dataset has been examined for estimating species richness by several researchers with different models, including the Poisson-lognormal model ([36]; 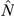 = 815±43), the Poisson-inverse Gaussian model ([37]; 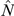 = 719), the Poisson-generalized inverse Gaussian model ([38]; 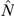 = 1000), and the mixed Gamma-Poisson model [18]. Chao and Bunge [18] extensively analyzed this dataset by using the cut-off point from *t* = 10 to 24 and compared six different estimators. They concluded that a stable value of 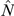 = 850 species was expected under the cut-off point below 24 (*t* 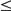
24). Like Chao and Bunge [18], we estimated population size using the same array of cut-off points. As summarized in Table 3 the estimate obtained from Model II, 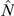 = 825 (the average over all cut-off points) is close to Bulmer's ([36]; 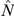 = 815) and Chao and Bunge's ([18]; 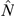 = 850) results.

**Table 3:** Estimates of species richness for Fisher's butterfly data [14] with Model II.

*t*	*n*	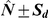	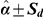	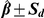
10	385	822.3±107.0	0.0334±0.0845	0.9318±0.1166
11	397	802.8±94.7	0.0598±0.0823	0.9895±0.1200
12	411	822.9±92.0	0.0440±0.0764	0.9474±0.1114
13	417	777.3±78.2	0.1030±0.0789	1.1060±0.1277
14	429	814.4±80.9	0.0618±0.0724	1.0109±0.1156
15	435	790.8±72.9	0.0946±0.0728	1.1122±0.1244
16	444	810.5±73.2	0.0732±0.0689	1.0667±0.1181
17	453	825.0±72.3	0.0592±0.0656	1.0324±0.1122
18	459	815.7±68.0	0.0736±0.0650	1.0826±0.1160
19	469	844.9±69.8	0.0415±0.0607	0.9908±0.1048
20	479	862.1±69.5	0.0260±0.0576	0.9369±0.0969
21	490	880.1±69.1	0.0111±0.0547	0.8786±0.0887
22	495	856.1±63.1	0.0416±0.0553	0.9598±0.0951
23	498	835.6±58.6	0.0692±0.0564	1.0516±0.1039
24	501	825.6±56.0	0.0841±0.0568	1.1145±0.1104

The same array of surveys (*t*) as Chao and Bunge [18], with *t* changing from 10 to 24, was used to estimate *N*.

The second example is the experimental cottontail abundance determined from two sets of live trapping data with known population sizes. The first dataset was collected in the Olentangy Wildlife experimental Station, Delaware County, Ohio, in 1961[39]. The second dataset was collected in 1963 at Robert Allerton Park, Monticello, Illinois. In the first dataset (Ohio), the observed capture-recapture frequencies from *f_1_* to *f*_7_ were 43, 16, 8, 6, 0, 2, and 1. This dataset was also examined by several researchers using different models, including Schnabel's estimate [40], Schumacher and Eschmeyer's method [41], MLE and the regression method based on the geometric model [39], and Chao-1 estimator [10]. The results obtained from both the regression method based on the geometric model and Chao's non-parameter estimator (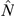 = 133.8±24.0 for Chao-1 estimator; [35]) are consistent with the actual population size. Analysis with Model II produces a negative α estimate, demonstrating a poor fit to the capture-recapture frequency pattern assumed in Model II. Analysis with Model I produces MLE 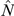 = 211.3±31.7 and 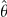 = 2.49±0.52. 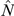 is overestimated (actual value *N* = 135) because of a low heterogeneity (the coefficient of variation (CV) for the low captured individuals = 0.619; [42]). This indicates that the actual capture probability distribution in this population (a low heterogeneity and a small population size) is biased from 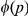 assumed in Model I (a large population and a high heterogeneity, say CV>0.8; [35]). In the second dataset (Illinois), the observed capture-recapture frequencies from *f_1_* to *f*_6_ were 36, 15, 13, 3, 1, and 1. Chao-1 estimator gives 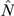 = 112.2±19.4 with a low to moderate heterogeneity (CV = 0.382). Model II produces 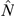 = 136.9±47.6, 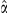 = 0.55±0.78, and 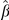 = 3.57±2.13, which is fairly close to the actual population size (*N* = 130; [39]).

### Applications to Ground Beef Batches

We now apply the proposed models to estimate the number of unique animals in ground beef batches (one batch is considered as one population). We had 57 time sequenced ground beef samples (each sample ∼250 g) taken from six 1 tonne batches from a single manufacturing line during a single production shift. There are 10 samples, analogous to the field surveys (sampling with replacement) in animal ecology [4], from Batches I to IV (manufacturing ID: 5.2, 5.3, 5.7, and 5.9), 9 samples from Batch V (ID: 5.11), and 8 samples from Batch VI (ID: 5.13). In each sample, we dissected 94 individual muscle fiber sub-samples, yielding 752∼940 sub-samples, extracted DNA, and genotyped over 30 SNP markers (∼160,000 genotypes in total). Missing genotypes were marked but excluded in analysis.

Several methods were applied to estimating the unique number of animals in individual batches and samples. One is the use of Genecap [43] where pairwise matching probabilities, in terms of the probability of identity (PI) were calculated assuming both Hardy-Weinberg equilibrium (HWE) for genotypic frequencies and linkage equilibrium. HWE was tested using Genepop software [44], showing that 6 out of 180 tests (∼3% in total) were not in HWE (see results below). Linkage disequilibria (LD) for all pairwise SNPs in each batch were tested using Genepop software as well, showing that all used SNPs were essentially in linkage equilibrium (see results below). The average multilocus PI in each batch is much smaller than 10^−5^ by using 25–30 SNP markers, which ensures the appropriate use of these markers for identifying individuals for estimating population size (mark-recapture method) [45], [46],[47], [48]. The modified Lincoln–Petersen method with the assumption of equal capture probability (homogeneous) was used to estimate population size *N* [4]. Each batch was separated in half for estimating recapture frequencies between two pooled samples. Population size *N* and its variance are calculated by 
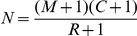
 and 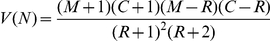, where *M* is the total number of animals captured and marked in one pooled sample, *C* is the total number of animals captured in the second pooled sample, and *R* is the number of animals recaptured in the second pooled sample.

In order to apply the proposed models to estimating *N*, we need to calculate the observed capture-recapture frequencies, *f_i_*'s (*i* = 1,…,*t*). The following steps were conducted. First, we identified the number of unique animals based on the statistical test (Pearson's correlation with student's t-test) of multilocus genotype matches with 30 SNP genotypes, removing the HWE assumption for calculating PI. Note that all pairs of SNPs were essentially independent from each other in each batch (see LD tests below). In order to identify unique individuals in a given sample, the individual SNP genotypes were transformed into numerical values. For example genotypes AA, AT, and TT were assigned 2, 1, and 0, respectively. Missing genotypes were designated another number and removed from the calculation. Pearson's correlation for each pair of individuals was tested using the significant level by Bonferroni correction (the type I error for the entire test was controlled at 1%). Two individuals are considered to be identical when they matched exactly, and replicates were removed from the analysis. Second, using the above described method, we identified the number of unique animals in each batch, i.e. *n* ( = 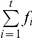) in the proposed model, by pooling all *t* samples that consisted of unique individuals. Third, using the same method as in the first step, we compared each of the *t* samples with the batch population (*n* individuals) and calculated the capture-recapture frequency of each of the *n* individuals in the batch, i.e. the estimates of *f_i_* (*i* = 1,…, *t*). In fact, our observations indicated that all exactly matched individuals (within or among samples) in our data sets of this study were identical in each of all genotypes (Pearson's correlation coefficients = 1.0). Once the observed frequencies ( *f_i_*'s) are available, the proposed models are then applied for estimating *N*. Two programs were written in SAS codes for this purpose and are available upon request from Hu.

As references, additional non-parameter estimators for unequal capture probability models were also applied, including Chao-1 estimator [10], the abundance-based coverage estimator (ACE) [12] and the first- and second-order jackknife estimators [8]. MLE based on the mixed Gamma-Poisson model was employed where *f_i_* (*i* = 1, 2, …, *t*) was assumed to follow Poisson distribution while *p* in 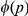 was assumed to follow a gamma distribution [18]. To measure the degree of heterogeneity among capture probabilities, the coefficient of variation (CV) for the low captured individuals was calculated (for formula, see [42]). Population size *N* with all these non-parameter estimators and Chao and Bunge's estimator can be estimated using SPADE software [35].

## Results

Population genetic analysis indicates that gene diversity ( = 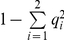, 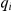 is the frequency of the *i*th allele at a SNP site) was about 0.46 per SNP for all six batches (Table 4). Among the total of 180 tests of the selected 30 SNPs in all batches, only six tests were in Hardy-Weinberg disequilibrium (Table 4; *P*-value<0.0003), indicating that most batches were essentially in HWE. Batch-based LD tests indicate that only two pairs of SNPs in Batch 6 (SNPs 14 and 19, SNPs 21 and 24; *P*-value<2.2
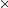
10^−5^) were in LD. Thus, SNP-17 in Batch 3, SNP-19 in all six batches, and SNP-21 were removed for further analyses. All SNPs eventually used in this study were independent from each other and in HWE.

**Table 4:** Gene diversity (*H*) and *P*-values (*P*) for statistically testing Hardy-Weinberg disequilibrium in six ground meat batches.

SNPs	Batch 1		Batch2		Batch 3		Batch 4		Batch 5		Batch 6	
	*H*	*P*	*H*	*P*	*H*	*P*	*H*	*P*	*H*	*P*	*H*	*P*
1	0.479	0.691	0.487	0.901	0.489	0.128	0.497	0.726	0.492	0.209	0.498	0.431
2	0.416	0.761	0.405	0.567	0.437	0.036	0.399	0.001	0.394	0.193	0.402	0.738
3	0.488	0.242	0.491	0.464	0.482	0.704	0.498	0.151	0.498	0.133	0.500	0.045
4	0.439	0.236	0.416	0.777	0.403	0.301	0.411	0.085	0.393	0.202	0.383	0.385
5	0.456	0.093	0.447	0.195	0.455	0.147	0.427	0.132	0.416	0.010	0.426	0.061
6	0.459	0.484	0.468	0.452	0.473	0.527	0.460	0.379	0.456	0.100	0.448	1.000
7	0.496	0.795	0.499	0.808	0.500	0.559	0.501	0.637	0.501	0.601	0.500	1.000
8	0.421	0.763	0.402	0.117	0.407	0.150	0.443	0.792	0.390	0.631	0.419	0.868
9	0.495	0.606	0.496	0.907	0.501	0.200	0.497	0.132	0.501	0.047	0.501	1.000
10	0.498	0.070	0.501	0.729	0.481	0.804	0.494	0.023	0.497	0.312	0.472	0.327
11	0.500	1.000	0.501	0.487	0.488	0.477	0.501	0.817	0.500	0.703	0.501	0.702
12	0.477	0.786	0.461	0.614	0.498	0.188	0.483	0.809	0.486	0.366	0.487	0.219
13	0.455	0.198	0.472	0.266	0.415	1.000	0.413	1.000	0.441	0.157	0.446	0.647
14	0.406	0.436	0.390	0.292	0.397	0.747	0.409	0.185	0.415	0.051	0.438	0.363
15	0.455	0.484	0.435	0.410	0.439	0.323	0.460	0.441	0.448	0.204	0.474	0.774
16	0.500	0.373	0.501	0.232	0.498	0.191	0.500	1.000	0.500	1.000	0.501	0.074
17	0.474	0.009	0.463	0.374	0.493	**0.000**	0.462	0.009	0.454	0.405	0.464	0.007
18	0.501	0.073	0.498	0.356	0.500	0.058	0.499	0.726	0.497	0.540	0.497	0.505
19	0.492	**0.000**	0.471	**0.000**	0.490	**0.000**	0.477	**0.000**	0.469	**0.000**	0.488	0.018
20	0.491	0.896	0.500	0.806	0.491	0.814	0.497	0.908	0.500	0.625	0.499	1.000
21	0.501	0.199	0.495	0.098	0.491	0.003	0.501	0.019	0.497	0.270	0.500	0.237
22	0.488	0.432	0.495	0.119	0.492	0.401	0.491	0.004	0.494	0.622	0.498	0.789
23	0.420	0.355	0.427	0.889	0.438	0.000	0.468	0.699	0.478	0.701	0.474	0.676
24	0.492	0.601	0.485	0.024	0.500	0.074	0.489	1.000	0.484	0.798	0.498	0.017
25	0.312	0.407	0.302	0.165	0.270	0.016	0.249	1.000	0.283	0.654	0.263	0.208
26	0.483	0.354	0.475	0.703	0.493	0.629	0.474	0.328	0.460	0.286	0.490	0.343
27	0.432	0.878	0.458	0.620	0.403	0.375	0.430	1.000	0.439	0.486	0.417	0.877
28	0.413	0.877	0.398	0.881	0.395	0.771	0.430	0.179	0.427	0.087	0.434	0.168
29	0.497	0.056	0.499	1.000	0.499	0.821	0.499	0.819	0.495	0.027	0.485	0.891
30	0.472	0.286	0.468	0.459	0.472	0.190	0.467	0.211	0.474	0.348	0.475	0.779
Average	0.464		0.460		0.460		0.461		0.459		0.463	

Table 5 summarises the observed capture-recapture frequencies, *f_i_*'s(*i* = 1,…,*t*), in all six batches, showing that all batches except Batch 1 displayed a highly skewed distribution of capture-recapture frequencies. CV estimates were 0.586, 0.893, 1.255, 0.836, 1.003, and 0.732 for Batches 1, 2, 3, 4, 5, and 6, respectively, indicating a high heterogeneity in capture probability in Batches 2, 3, 4, and 5 (CV>0.8), but not in Batches 1 and 6 (CV<0.8; [35]). As expected, Lincoln–Petersen's estimator severely underestimated population size due to the presence of heterogeneous capture probability in each batch that violated the assumption of homogeneous capture probability in this method. As suggested by Chao and Shen [35], the Chao-1 estimator (for a low to moderate heterogeneity in capture probability) produced the lower bound estimates of population size, but its estimates were greater than those obtained with Lincoln-Petersen's estimator. The first- and second-order jackknife estimators provided comparable estimates to Chao-1 estimator. Chao and Shen [35] recommended the use of ACE-1 for the population with a high heterogeneity (CV>0.8) since this estimator uses the information on a highly heterogeneous capture probability in estimation. The ACE-1 estimator produced higher estimates of population size for Batches 2, 3, 4, and 5, 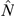 = 576.8∼1011.3, but not for Batches 1 and 6. Batch 3 had the largest population size, followed by Batch 5, which was consistent with the rank of CV values.

**Table 5:** Estimates of the number of animals in different ground meat batches (point estimates ± standard errors).

Model	Capture-recapture frequency	Batch 1	Batch 2	Batch 3	Batch 4	Batch 5	Batch 6
	*f*_1_	94	159	199	164	186	142
	*f*_2_	59	59	49	61	49	53
	*f*_3_	44	40	25	47	17	22
	*f*_4_	26	20	11	12	9	10
	*f*_5_	11	7	2	7	5	3
	*f*_6_	4	6	6	6	0	3
	*f*_7_	6	3	2	3	3	1
	*f*_8_	1	2	1	3	0	0
	*f*_9_	2	2	2	0	0	
	*f*_10_	0	0	0	0		
Lincoln–Petersen method		291±33	419±57	491±85	427±60	453±87	365±23
Chao-1		321.9±20.2	512.2±46.3	701.1±83.8	523.5±46.9	622.0±74.7	424.2±43.5
1st order jackknife		340.8±13.7	456.7±17.8	495.6±19.9	466.7±18.1	454.6±19.3	375.6±16.8
2nd order jackknife		375.8±23.7	556.5±30.8	645.1±34.5	569.5±31.3	591.0±33.3	464.3±29.1
ACE-1		331.6±21.6	576.8±62.1	1011.3±169.8	577.9±60.2	823.5±136.8	484.8±62.8
Gamma-Poisson-MLE		335.9±27.1	821.8±287.3	not convergent	771.4±231.5	not convergent	667.7±264.7
Proposed model-MLE		411.4±56.3 (*α* = 0.67±0.29, *β* = 3.69±0.86)	1042.6±80.1 (*θ* = 2.77±0.29)	1298.8±113.7 (*θ* = 4.31±0.49)	1111.0±86.4 (*θ* = 2.62±0.27)	1366.8±135.4 (*θ* = 4.23±0.52)	1010.8±99.3 (*θ* = 3.37±0.42)

The mixed Gamma-Poisson model [18] provided larger estimates of population size for Batches 2 (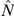 = 821.8±287.3), 4(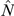 = 771.4±231.5), and 6 (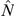 = 667.7±264.7). Iterations were not convergent for Batches 3 and 5 due to the high heterogeneity in capture probability (Table 5).

With application of the proposed models in this study, we first applied Model II to obtain MLE of *N*, *α*, and *β* because Model I is the specific case of Model II. If the estimate of α is negative, we then apply Model I. Results indicate that α estimates were negative in all batches except Batch 1. Thus, we used Model II to analyze Batch 1 data and Model I to analyze the other batches. The population size in Batch 1 was 411.4±56.3, but the 95%CI (confidence interval) overlapped with the 95%CI obtained from the second jackknife estimator. The average population sizes were greater than 1000 (1011∼1367) in the remaining batches. Since a very high heterogeneity in capture probability exists in Batches 2–6, all the examined non-parameter methods produce severe underestimates of population [35], as indicated from the simulation results in the preceding section. The capture probability distributions in these batches more likely follows the assumption of 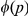 in Model I, and the estimates of population size are close to their actual sizes (see simulation results for *N* = 1000 and *t*>6 in Table 1). Estimates in Batches 2, 4, and 6 with Model I were mainly distributed within the 95%CI obtained from the mixed Gamma-Poisson estimator. Estimates, 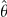
's, were positively related to the CV values, reflecting the extent of heterogeneity in capture probability.

## Discussion

In this study, we proposed two related statistical models for estimating the number of animals in a population. One uses a model similar to the modified continuous version of Fisher's logarithmic series model to describe capture probability density function 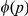 (Model I); while the other uses the modified beta function to describe 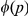 (Model II). Model I is the specific case of Model II. In each model, the lower bound for capture probability is truncated by 1/*N*, and this lower bound approaches zero as the population size increases. This way of removing the non-captured probability is more meaningful since the capture probability for each individual must be nonzero in biology (each individual must be obtainable in theory) although the lower bound may be allowed to be zero in statistics. The idea is different in biological meaning from Wright's thinking in calculating the existent alleles in a population ([29], p 398) or a similar way in calculating existent individuals in community ecology [22], [30] for the abundance data. Good ([26], pp 251–252) discussed the truncated distribution related to Model I for the abundance data, but did not discuss how to determine the lower bound. In general, Model I is suitable for the population with a very high heterogeneity in capture probability (say, CV>0.8) and a large population size; while Model II is suitable for the population with a relatively lower heterogeneity in capture probability (say, a moderate heterogeneity; [35]) and a relatively smaller population size. Both Models I and II provide new additions to the incidence-based methods of estimating population size.

Selection of appropriate model is important for analyzing empirical data since each model has its own strength and limitation. Estimates of population size for parametric models are sensitive to model assumptions about the capture probability density distribution [21]. Bunge and Barger [17] reviewed several parametric models for the abundance data and discussed the connection between abundance and incidence models. Our proposed two models are based on incidence data samples. The strength of Model I is its suitability to the population of a very high heterogeneity in capture probability and its better performance over the existing non-parameter estimators. One caution is that a slightly positive bias for the mean estimate may occur although the actual parameters are not significantly different from estimates (the actual values are within the ranges of one standard deviation). The weakness of Model I is that a substantially biased estimate can be produced when the heterogeneity in capture probability is low or moderate, as indicated from the example of experimental cottontail abundance. Model II has comparable performances with other existing non-parameter models in the presence of a relatively lower heterogeneity in capture probability or in the case of capturing a large proportion of population. The setting of 1/*N* as the lower bound predicts a better performance of Model II for a large population, as indicated from the example of Fisher's butterfly datasets.

It is important to understand that many distinct processes may be involved in generating a highly heterogeneous capture probability in a single manufacturing batch. Most meat in a ground beef batch comes from off-cuts or trimmings. These raw materials are usually blended during processing as it would be entirely impractical and uneconomic to process, label or tag each component separately [49]. Because different animals exhibit wide variation in meat and fat content, the quantity and quality of trimmings varies considerably among animals. Thus, different animals have quite variable contributions to a single beef batch. This forms the biological basis for generating heterogeneous capture probability although sampling process or animal behaviours could likely modify 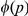. Many thousands of animals are processed per day in large scale slaughterhouses, and this may subsequently result in a large number of animals in a single grind batch. In addition, the number of animals in a single batch is affected by several factors in the supply chain, including the specific grind manufacturing process, the number of diverse farms providing cattle to the processors, the scale of production and the use of lean finely textured beef (LFTB). These processes could explain the highly skewed pattern of capture-recapture frequencies in the five batches. Many animals can be captured with a low frequency (e.g., once) and a few animals can be captured multiple times.

The observed capture-recapture frequencies, *f_i_*'s (*i* = 1,…, *t*), in six manufacturing batches indicate a high heterogeneity in capture probability in a single ground beef batch. A highly skewed opposite J-shape in five batches (Batches 2–6) implies that a potentially large number of individuals are present in them. An average of 411 to1367 animals was present in the six grind batches. These estimates indicate high variation in the number of animals among different batches from the same manufacturer on a single production line during a single production shift. From the manufacturing records, the batches examined here were compounded from raw materials consisting of 3 grades of fresh and frozen beef trim with unequal weights of components among batches. In addition up to 10% of each batch was comprised of LFTB and rework. Animal abundance in each raw material is unknown *a priori*. The estimates derived here are informative as a reference in decision-making in the case of food safety recalls.

It is of interest to compare the similarity and difference in mark-recapture experiments between the conventional field of animal ecology [4] and the laboratory or non-invasive DNA-profile detection in a ground beef batch. Both animal abundance and habitats/behaviours can affect the capture probability distribution 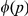 in field animal surveys. With the ground beef batch, population composition can affect the heterogeneity in capture probability if the samples for DNA profile testing are randomly taken. Further, use of DNA profiles to identify individuals can result in false positive capture if the number of markers is small [46]. One striking difference is that multiple copies of the same animal can occur in one survey in a grind batch, but infrequently take place in the field animal survey. The marked animals are not recorded twice in a single survey. In a single grind batch, the same DNA profiles from different parts of one animal could be sampled, similar to DNA samples from multiple shed hair samples of animals [50]. Thus, to employ the standard mark-recapture method, the duplicated DNA profiles must be removed in a single survey. Lukas et al. [50] proposed an alternative likelihood function that can use the duplicated DNA profiles in a single survey, but the proposed algorithm is too complex for application.

So far, the mechanisms for generating the pattern of capture probability distribution 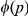 have not been fully examined except for the application of Fisher's logarithmic series [18]. Also, the conventional mark-recapture framework has not been linked to the relevant biological mechanisms for maintaining a closed population and the relationship between the capture probability distribution and population composition or animal activities. In most situations, the assumption of a closed population holds in multiple field surveys within short-time intervals (no change of population size through births, deaths, immigration and emigration). Fisher's logarithm series model or the more explicitly continuous version indeed refers to the case of neutral metacommunity or completely isolated community with a fixed size [28], [30]. The capture probability distribution in Model I reflects the pattern in a closed population. Unlike Model I, Model II is probably more flexible for a closed population or an open population (e.g., the carry-over between batches in the same manufacturer) with a fixed population size *N*. Previous theories in population genetics demonstrate that the beta function can be used to describe the distribution of gene frequency (abundance data) in a local open population with a fixed size ([29], p 362), given the presence of a constant ratio of effective (*N_e_*) versus real (*N*) population sizes. Bunge and Barger [17] have discussed the connection of the beta-distribution for incidence model to the log-beta distribution for the abundance models. Such a connection needs further exploration from the zero-truncated beta function for incidence model to the function for the abundance model. It cannot be excluded that exchanges of individuals may generate an array of patterns of capture probability distributions in an open population (Figure 1B). Different from the model of Jolly [51] and Seber and Manly [52], Model II can deal with the case of heterogeneous capture probability. Previous models for an open population assumed constant homogeneous capture probability [5], [53], but their comparisons with Model II need empirical evaluations.

To apply the proposed models for estimating animal abundance in a single batch, the following steps are needed. First, we need to select appropriate markers to identify individual profiles. For a single marker, a large gene diversity or heterozygosity should be selected. For multiple markers, linkage equilibria among them should be required so as to avoid redundant information. The number of markers can be decided by their joint PI (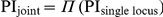), or more conservatively by the joint PI of sibs as the reference. Waits et al. [48] suggested that the number of markers generating a joint PI<0.0001 can be used for mark-recapture analysis. The present study sufficiently meets these two criteria. Second, we need to decide an appropriate survey scheme. Our simulation results recommend that the scheme of multiple surveys, each with a relatively small sample size, is better than the scheme with limited surveys, each with a relatively large sample size. Multiple surveys with small sample sizes are better in reflecting the true pattern of capture-recapture frequency. However, this is not the case for the non-parameter estimators that rely on the frequencies of one- and two-time captures (e.g., *f*_1_ and *f*_2_ in Chao's estimator [10]). Third, the capture-recapture frequencies, *f_i_*'s (*i* = 1,…, *t*), can be calculated by either Genecap (HWE and without LD; [43]) or the Pearson's coefficients (without LD) used in this study. Fourth, once all capture and recapture frequencies ( *f_i_*'s) are available, MLE can be obtained by applying the proposed models. The advantage of the proposed model over some non-parameter models lies in that the full information on capture-recapture frequency is employed. Further, MLE becomes unbiased as the total number of captured individuals (*n*) increases in multiple surveys.

Finally, in the phase of meat processing, tracing the finished ground meat products inevitably involves decision-making on tracing within and between batches. Our results recognize the complexity of tracking and tracing ground meat batches based on the trimmings since more than 1000 animals could be included in a single grind batch. Grinding operations are the last phase before the market or end-users in the meat supply chain [49]. The existing meat traceability systems are primarily documented in regards to the primal cuts [54] and have inadequate tracing of the mixed trimmings. Also, analysis with Genepop indicates that population (batch) differentiation was very small among these six batches, with the 95%CI for multilocus *F_st_* being within [0.1%, 0.2%] (detailed results not shown here). Further extensive analysis is needed to investigate batch differentiation using measures differing in sensitivity to population differentiation. With the use of Models I and II, a large number of animals comprise each batch of ground meat. Based on this premise, a sampling scheme can be implemented which provides sufficient DNA information to effectively differentiate ground meat batches. Development of additional statistical models to establish a reliable framework for the genetic characterization of individual ground beef batches is undertaken. Establishing methods by which individual ground beef batches can be identified can significantly reduce the scope of a product recall in the event of a contamination incident. For instance, contamination with E. coli 0157:H7 accounts for 24% of FSIS recalls in the United States in 2009 [55].This would have a significant impact on the economics and efficiency of the recall process.

## Supporting Information

Appendix S1 **Partial differentials of the log likelihood function for Model I.**
(DOC)

Appendix S2 **Partial differentials of the log likelihood function for Model II.**
(DOC)
