# Supplementary material for: Estimating Animal Abundance in Ground Beef Batches Assayed with Molecular Markers
Source: PLoS One. 2012 Mar 30;7(3):e34191. doi: 10.1371/journal.pone.0034191 (PMC3316629; doi:10.1371/journal.pone.0034191)
Supplement: Appendix S1 — Partial differentials of the log likelihood function for Model I. (DOC) [file pone.0034191.s001.doc]

**Appendix S1: Partial differentials of the log likelihood function for Model I**

From the likelihood function, equation (1), in the main text, the partial differentials of the log likelihood function $\ln L$ with respect to *N* and $\theta$ are derived as

$$\frac{\partial\ln L}{\partial N}=\psi(N+1)-\psi(N-n+1)+\ln\pi_{0}(\varphi)+(N-n)\frac{\partial\ln\pi_{0}(\varphi)}{\partial N}+\sum_{i=1}^{t} f_{i}\frac{\partial\ln\pi_{i}(\varphi)}{\partial N}$$

=$\psi(N+1)-\psi(N-n+1)+\ln\pi_{0}(\varphi)-\frac{1}{r_{1}(0,0,\theta,N)}\left(1-\frac{1}{N}\right)^{\theta-1}+(N-n)\frac{1}{r_{1}(0,t,\theta,N)}\frac{1}{N}\left(1-\frac{1}{N}\right)^{t+\theta-1}$

$+\sum_{i=1}^{t} f_{i}\frac{1}{r_{1}(i,t,\theta,N)}\frac{1}{N^{i+1}}\left(1-\frac{1}{N}\right)^{t+\theta-i-1}$. (A1)

$\frac{\partial\ln L}{\partial\theta}=(N-n)\left(\frac{r_{2}(0,t,\theta,N)}{r_{1}(0,t,\theta,N)}-\frac{r_{2}(0,0,\theta,N)}{r_{1}(0,0,\theta,N)}\right)+\sum_{i=1}^{t} f_{i}\left(\frac{r_{2}(i,t,\theta,N)}{r_{1}(i,t,\theta,N)}-\frac{r_{2}(0,0,\theta,N)}{r_{1}(0,0,\theta,N)}\right)$. (A2)

During the calculation, the digamma function can be numerically approximated by the following formula with a sufficient accuracy$\psi(x)=\ln(x)-1/(2x)-1/(12x^{2})+1/(120x^{4})-1/(252x^{6})$ (Bernardo, 1976).

The second-order partial differentials are derived as:

$$+\sum_{i=1}^{t} f_{i}\left(-\frac{1}{r_{1}^{2}(i,t,\theta,N)}\frac{1}{N^{2(i+1)}}\left(1-\frac{1}{N}\right)^{2(t+\theta-i-1)}-\frac{i+1}{r_{1}(i,t,\theta,N)}\frac{1}{N^{i+2}}\left(1-\frac{1}{N}\right)^{t+\theta-i-1}+\frac{t+\theta-i-1}{r_{1}(i,t,\theta,N)}\frac{1}{N^{i+3}}\left(1-\frac{1}{N}\right)^{t+\theta-i-2}\right)$$

$$-\left(-\frac{1}{r_{1}^{2}(0,0,\theta,N)}\frac{1}{N}\left(1-\frac{1}{N}\right)^{2(\theta-1)}-\frac{1}{r_{1}(0,0,\theta,N)}\frac{1}{N}\left(1-\frac{1}{N}\right)^{\theta-1}+\frac{\theta-1}{r_{1}(0,0,\theta,N)}\frac{1}{N^{2}}\left(1-\frac{1}{N}\right)^{\theta-2}\right)$$

$+(N-n)\left(-\frac{1}{r_{1}^{2}(0,t,\theta,N)}\frac{1}{N^{2}}\left(1-\frac{1}{N}\right)^{2(t+\theta-1)}-\frac{1}{r_{1}(0,t,\theta,N)}\frac{1}{N^{2}}\left(1-\frac{1}{N}\right)^{t+\theta-1}+\frac{\theta-1}{r_{1}(0,t,\theta,N)}\frac{1}{N^{3}}\left(1-\frac{1}{N}\right)^{t+\theta-2}\right)$. (A3)

$$\frac{\partial^{2}\ln L}{\partial\theta\partial N}=\frac{r_{2}(0,t,\theta,N)}{r_{1}(0,t,\theta,N)}-\frac{r_{2}(0,0,\theta,N)}{r_{1}(0,0,\theta,N)}$$

$$+\left(1-\frac{n}{N}\right)\left(1-\frac{1}{N}\right)^{t+\theta-1}\left(\frac{1}{r_{1}(0,t,\theta,N)}\ln(1-\frac{1}{N})-\frac{r_{2}(0,t,\theta,N)}{r_{1}^{2}(0,t,\theta,N)}\right)$$

$$+\sum_{i=1}^{t} f_{i}\frac{1}{N^{i+1}}\left(1-\frac{1}{N}\right)^{t+\theta-i-1}\left(\frac{1}{r_{1}(i,t,\theta,N)}\ln(1-\frac{1}{N})-\frac{r_{2}(i,t,\theta,N)}{r_{1}^{2}(i,t,\theta,N)}\right)$$

$-\left(1-\frac{1}{N}\right)^{\theta-1}\left(\frac{1}{r_{1}(0,0,\theta,N)}\ln(1-\frac{1}{N})-\frac{r_{2}(0,0,\theta,N)}{r_{1}^{2}(0,0,\theta,N)}\right)$. (A4)

$$\frac{\partial^{2}\ln L}{\partial\theta^{2}}=(N-n)\left(\frac{r_{3}(0,t,\theta,N)}{r_{1}(0,t,\theta,N)}-\frac{r_{2}^{2}(0,t,\theta,N)}{r_{1}^{2}(0,t,\theta,N)}\right)$$

$$+\sum_{i=1}^{t} f_{i}\left(\frac{r_{3}(i,t,\theta,N)}{r_{1}(i,t,\theta,N)}-\frac{r_{2}^{2}(i,t,\theta,N)}{r_{1}^{2}(i,t,\theta,N)}\right)$$

$-N\left(\frac{r_{3}(0,0,\theta,N)}{r_{1}(0,0,\theta,N)}-\frac{r_{2}^{2}(0,0,\theta,N)}{r_{1}^{2}(0,0,\theta,N)}\right)$. (A5).

Similarly, the trigamma function is numerically approximated by the following formula during the calculation:

$\psi'(x)=1/x+1/(2x^{2})+1/(6x^{3})-1/(30x^{5})+1/(42x^{7})$(Abramowitz and Stegun, 1972).

References

Abramowitz M, Stegun IA (1972) Psi (digamma) function. In: Handbook of Mathematical Functions with Formulas, Graphs, and Mathematical Tables, 9th printing. New York: Dover. pp. 258-259.

Bernardo JM (1976) Algorithm as 103: Psi (digamma) function. *Journal of the Royal Statistical Society. Series C* (*Applied Statistics*) 25(3): 315-317.
