# Supplementary material for: Estimating Animal Abundance in Ground Beef Batches Assayed with Molecular Markers
Source: PLoS One. 2012 Mar 30;7(3):e34191. doi: 10.1371/journal.pone.0034191 (PMC3316629; doi:10.1371/journal.pone.0034191)
Supplement: Appendix S2 — Partial differentials of the log likelihood function for Model II. (DOC) [file pone.0034191.s002.doc]

**Appendix S2: Partial differentials of the log likelihood function for Model II**

According to the likelihood function in the main text, the partial differentials of the log likelihood function $\ln L$ with respect to *N*, $\alpha$, and $\beta$are derived as

$$\frac{\partial\ln L}{\partial N}=\psi(N+1)-\psi(N-n+1)+\ln\pi_{0}(\varphi)-\frac{1}{R_{1}(0,0,\alpha,\beta,N)}\frac{1}{N^{\alpha}}\left(1-\frac{1}{N}\right)^{\beta-1}+(N-n)\frac{1}{R_{1}(0,t,\alpha,\beta,N)}\frac{1}{N^{\alpha+1}}\left(1-\frac{1}{N}\right)^{t+\beta-1}$$

$+\sum_{i=1}^{t} f_{i}\frac{1}{R_{1}(i,t,\alpha,\beta,N)}\frac{1}{N^{\alpha+i+1}}\left(1-\frac{1}{N}\right)^{t+\beta-i-1}$. (B1)

$\frac{\partial\ln L}{\partial\alpha}=(N-n)\left(\frac{R_{2a}(0,t,\alpha,\beta,N)}{R_{1}(0,t,\theta,N)}-\frac{R_{2a}(0,0,\alpha,\beta,N)}{R_{1}(0,0,\alpha,\beta,N)}\right)+\sum_{i=1}^{t} f_{i}\left(\frac{R_{2a}(i,t,\alpha,\beta,N)}{R_{1}(i,t,\theta,N)}-\frac{R_{2a}(0,0,\alpha,\beta,N)}{R_{1}(0,0,\alpha,\beta,N)}\right)$. (B2)

$\frac{\partial\ln L}{\partial\beta}=(N-n)\left(\frac{R_{2b}(0,t,\alpha,\beta,N)}{R_{1}(0,t,\theta,N)}-\frac{R_{2b}(0,0,\alpha,\beta,N)}{R_{1}(0,0,\alpha,\beta,N)}\right)+\sum_{i=1}^{t} f_{i}\left(\frac{R_{2b}(i,t,\alpha,\beta,N)}{R_{1}(i,t,\theta,N)}-\frac{R_{2b}(0,0,\alpha,\beta,N)}{R_{1}(0,0,\alpha,\beta,N)}\right)$. (B3)

The second-order partial differentials are derived as:

$$+\sum_{i=1}^{t} f_{i}\left(-\frac{1}{R_{1}^{2}(i,t,\alpha,\beta,N)}\left(\frac{1}{N}\right)^{2(\alpha+i+1)}\left(1-\frac{1}{N}\right)^{2(t+\beta-i-1)}-\frac{\alpha+i+1}{R_{1}(i,t,\alpha,\beta,N)}\left(\frac{1}{N}\right)^{\alpha+i+2}\left(1-\frac{1}{N}\right)^{t+\beta-i-1}+\frac{t+\beta-i-1}{R_{1}(i,t,\alpha,\beta,N)}\left(\frac{1}{N}\right)^{\alpha+i+3}\left(1-\frac{1}{N}\right)^{t+\beta-i-2}\right)$$

$$-\left(-\frac{1}{R_{1}^{2}(0,0,\alpha,\beta,N)}\frac{1}{N^{2\alpha+1}}\left(1-\frac{1}{N}\right)^{2(\beta-1)}-\frac{\alpha+1}{R_{1}(0,0,\alpha,\beta,N)}\frac{1}{N^{\alpha+1}}\left(1-\frac{1}{N}\right)^{\beta-1}+\frac{\beta-1}{R_{1}(0,0,\alpha,\beta,N)}\frac{1}{N^{\alpha+2}}\left(1-\frac{1}{N}\right)^{\beta-2}\right)$$

$+(N-n)\left(-\frac{1}{R_{1}^{2}(0,t,\alpha,\beta,N)}\frac{1}{N^{2(\alpha+1)}}\left(1-\frac{1}{N}\right)^{2(t+\beta-1)}-\frac{\alpha+1}{R_{1}(0,t,\alpha,\beta,N)}\frac{1}{N^{\alpha+2}}\left(1-\frac{1}{N}\right)^{t+\beta-1}+\frac{\theta-1}{R_{1}(0,t,\alpha,\beta,N)}\frac{1}{N^{\alpha+3}}\left(1-\frac{1}{N}\right)^{t+\beta-2}\right)$. (B4)

$$\frac{\partial^{2}\ln L}{\partial N\partial\alpha}=\frac{R_{2a}(0,t,\alpha,\beta,N)}{R_{1}(0,t,\alpha,\beta,N)}-\frac{R_{2a}(0,0,\alpha,\beta,N)}{R_{1}(0,0,\alpha,\beta,N)}$$

$$+\left(N-n\right)\frac{1}{N^{\alpha+1}}\left(1-\frac{1}{N}\right)^{t+\beta-1}\left(\frac{1}{R_{1}(0,t,\alpha,\beta,N)}\ln(\frac{1}{N})-\frac{R_{2a}(0,t,\alpha,\beta,N)}{R_{1}^{2}(0,t,\alpha,\beta,N)}\right)$$

$$+\sum_{i=1}^{t} f_{i}\left(\frac{1}{N}\right)^{\alpha+i+1}\left(1-\frac{1}{N}\right)^{t+\beta-i-1}\left(\frac{1}{R_{1}(i,t,\alpha,\beta,N)}\ln(\frac{1}{N})-\frac{R_{2a}(i,t,\alpha,\beta,N)}{R_{1}^{2}(i,t,\alpha,\beta,N)}\right)$$

$-\frac{1}{N^{\alpha}}\left(1-\frac{1}{N}\right)^{\beta-1}\left(\frac{1}{R_{1}(0,0,\alpha,\beta,N)}\ln(\frac{1}{N})-\frac{R_{2a}(0,0,\alpha,\beta,N)}{R_{1}^{2}(0,0,\alpha,\beta,N)}\right)$. (B5)

$$\frac{\partial^{2}\ln L}{\partial N\partial\beta}=\frac{R_{2b}(0,t,\alpha,\beta,N)}{R_{1}(0,t,\alpha,\beta,N)}-\frac{R_{2b}(0,0,\alpha,\beta,N)}{R_{1}(0,0,\alpha,\beta,N)}$$

$$+\left(N-n\right)\frac{1}{N^{\alpha+1}}\left(1-\frac{1}{N}\right)^{t+\beta-1}\left(\frac{1}{R_{1}(0,t,\alpha,\beta,N)}\ln(1-\frac{1}{N})-\frac{R_{2b}(0,t,\alpha,\beta,N)}{R_{1}^{2}(0,t,\alpha,\beta,N)}\right)$$

$$+\sum_{i=1}^{t} f_{i}\left(\frac{1}{N}\right)^{\alpha+i+1}\left(1-\frac{1}{N}\right)^{t+\beta-i-1}\left(\frac{1}{R_{1}(i,t,\alpha,\beta,N)}\ln(1-\frac{1}{N})-\frac{R_{2b}(i,t,\alpha,\beta,N)}{R_{1}^{2}(i,t,\alpha,\beta,N)}\right)$$

$-\frac{1}{N^{\alpha}}\left(1-\frac{1}{N}\right)^{\beta-1}\left(\frac{1}{R_{1}(0,0,\alpha,\beta,N)}\ln(1-\frac{1}{N})-\frac{R_{2b}(0,0,\alpha,\beta,N)}{R_{1}^{2}(0,0,\alpha,\beta,N)}\right)$. (B6)

$$\frac{\partial^{2}\ln L}{\partial\alpha\partial\beta}=(N-n)\left(\frac{R_{2ab}(0,t,\alpha,\beta,N)}{R_{1}(0,t,\alpha,\beta,N)}-\frac{R_{2a}(0,t,\theta,N)R_{2b}(0,t,\alpha,\beta,N)}{R_{1}^{2}(0,t,\alpha,\beta,N)}\right)$$

$$+\sum_{i=1}^{t} f_{i}\left(\frac{R_{2ab}(i,t,\alpha,\beta,N)}{R_{1}(i,t,\theta,N)}-\frac{R_{2a}(i,t,\alpha,\beta,N)R_{2b}(i,t,\alpha,\beta,N)}{R_{1}^{2}(i,t,\alpha,\beta,N)}\right)$$

$-N\left(\frac{R_{2ab}(0,0,\alpha,\beta,N)}{R_{1}(0,0,\alpha,\beta,N)}-\frac{R_{2a}(0,0,\theta,N)R_{2b}(0,0,\alpha,\beta,N)}{R_{1}^{2}(0,0,\alpha,\beta,N)}\right)$. (B7)

$$\frac{\partial^{2}\ln L}{\partial\alpha^{2}}=(N-n)\left(\frac{R_{3a}(0,t,\alpha,\beta,N)}{R_{1}(0,t,\alpha,\beta,N)}-\frac{R_{2a}^{2}(0,t,\theta,N)}{R_{1}^{2}(0,t,\alpha,\beta,N)}\right)$$

$$+\sum_{i=1}^{t} f_{i}\left(\frac{R_{3a}(i,t,\alpha,\beta,N)}{R_{1}(i,t,\theta,N)}-\frac{R_{2a}^{2}(i,t,\alpha,\beta,N)}{R_{1}^{2}(i,t,\alpha,\beta,N)}\right)$$

$-N\left(\frac{R_{3a}(0,0,\alpha,\beta,N)}{R_{1}(0,0,\alpha,\beta,N)}-\frac{R_{2a}^{2}(0,0,\theta,N)}{R_{1}^{2}(0,0,\alpha,\beta,N)}\right)$. (B8)

$$\frac{\partial^{2}\ln L}{\partial\beta^{2}}=(N-n)\left(\frac{R_{3b}(0,t,\alpha,\beta,N)}{R_{1}(0,t,\alpha,\beta,N)}-\frac{R_{2b}^{2}(0,t,\alpha,\beta,N)}{R_{1}^{2}(0,t,\alpha,\beta,N)}\right)$$

$$+\sum_{i=1}^{t} f_{i}\left(\frac{R_{3b}(i,t,\alpha,\beta,N)}{R_{1}(i,t,\theta,N)}-\frac{R_{2b}^{2}(i,t,\alpha,\beta,N)}{R_{1}^{2}(i,t,\alpha,\beta,N)}\right)$$

$-N\left(\frac{R_{3b}(0,0,\alpha,\beta,N)}{R_{1}(0,0,\alpha,\beta,N)}-\frac{R_{2b}^{2}(0,0,\theta,N)}{R_{1}^{2}(0,0,\alpha,\beta,N)}\right)$. (B9)
